# Regional Odontodysplasia Crossing Midline: A Rare Case Report

**DOI:** 10.5005/jp-journals-10005-1102

**Published:** 2010-04-15

**Authors:** Vinod Upadhyay, TP Chaturvedi, RK Pandey, Akhilanand Chaurasia, Parul Singh

**Affiliations:** 1 Senior Resident, Faculty of Dental Sciences, Banaras Hindu University, Varanasi, Uttar Pradesh, India; 2 Professor, Faculty of Dental Sciences, Banaras Hindu University, Varanasi, Uttar Pradesh, India; 3 Professor and Head, Department of Pedodontics, CSMMU, Lucknow, Uttar Pradesh, India; 4 Resident, Department of Pedodontics, CSMMU, Lucknow, Uttar Pradesh, India

**Keywords:** Regional odontodysplasia, Ghost teeth, Periapical inflammation.

## Abstract

Regional odontodysplasia is a nonhereditary, uncommon developmental abnormality of teeth. Females have more predilections for regional odontodysplasia. The enamel, dentin and pulp of teeth are affected and radiographically, teeth are described as “ghost teeth”. Many of these :eeth do not erupt and have an increased risk for caries and periapical inflammation. Since the literature on regional odontodysplasia is limited, here is need to discuss this anomaly to have a better approach for the diagnosis and treatment.

## INTRODUCTION

Regional odontodysplasia is a rare developmental anomaly involving both mesodermal and ectodermal components of teeth.^1^ This condition was first reported by Hitchin.^2^ The word “odontodysplasia” was coined by Zegarelli et al.^3^ This anomaly affects only one quadrant, “regional odontodysplasia” became the most accepted term to define it. Other denominations for the same condition are odontogenic dysplasia, localized arrest tooth development, ghost teeth, odontogenisis imperfecta, unilateral dental malformation and familial amelodentinal dysplasia. Regional odontodysplasia affects both primary and permanent denti-tions.^4^ The maxilla is affected twice as often as the mandible, where the maxillary left quadrant being the most commonly involved.^5^ Regarding the teeth, the central and lateral incisors are more frequently affected than the posterior teeth.^6^ It has been suggested that this condition is more common in girls than in boys.

## CASE REPORT

**Fig. 1:**
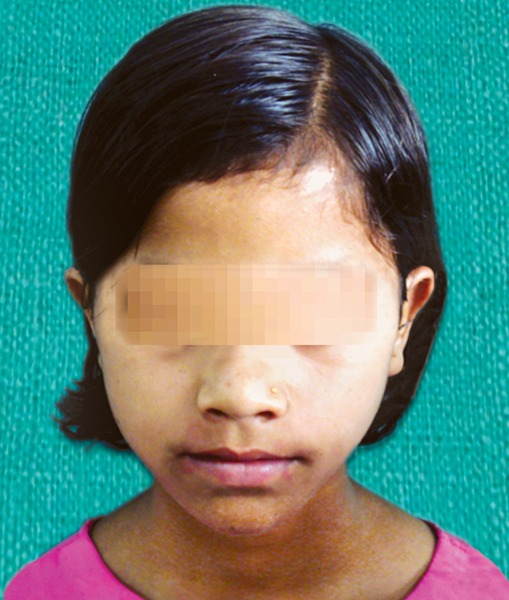
No facial asymmetry

**Fig. 2:**
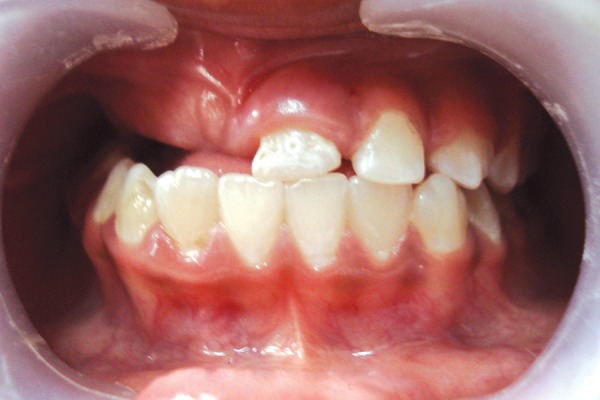
Intraoral view

**Fig. 3:**
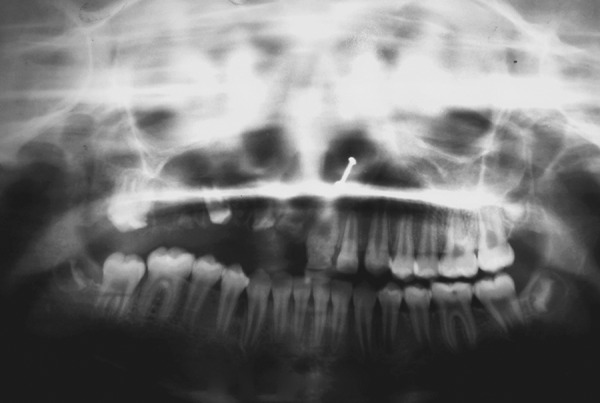
Panoramic view

A 13-year-old girl reported to Department of Pedodontics and Preventive Dentistry, CSMMU, Lucknow, with chief complaint of unerupted upper right side teeth. There was normal exfoliation of deciduous teeth. The patient’s medical history was nonsignificant. Parents reported no previous history of tooth or genetic anomalies on either side of the family. Extraoral examination revealed no facial asymmetry (Fig. 1). Intraoral examination revealed a full complement of dentition with normal occlusion except for the maxillary right quadrant. In right maxillary quadrant central incisor, lateral incisor, canine, first and second premolar, first and second molar were unerupted. The associated alveolar mucosa was slightly hyperplastic and covered by fibrous tissue. However, on left maxillary quadrant only central incisor was hypoplastic (Fig. 2). In the mandibular arch, full complement of teeth are present except third molar. Radiographic investigation was done using panoramic (Fig. 3) and periapical radiographs (Figs 4A and B). The mandibular dentition was normal as was the left maxillary dentition, except the maxillary left central incisor. The left maxillary central incisor showed wide pulp chamber with malformed crown. The right maxillary central and lateral incisor showed malformed crown structure and thin radi-opaque contours with no distinction between enamel and dentin with wide pulp chambers (Fig. 4A). The maxillary right canine showed unorganized crown with thin radiopaque outline of teeth given “ghost-like” appearance. The maxillary right first premolar did not look as affected when compare to other affected teeth. The maxillary right second premolar and maxillary right first molar showed unorganized crown with thin radiopaque outline of teeth given “ghost-like” appearance (Fig. 4B). The maxillary right second molar did not look as affected when compared to the other affected teeth (Fig. 3).

On the basis of the clinical and radiographic findings provisional diagnosis of regional odontodysplasia was made.

**Fig. 4A:**
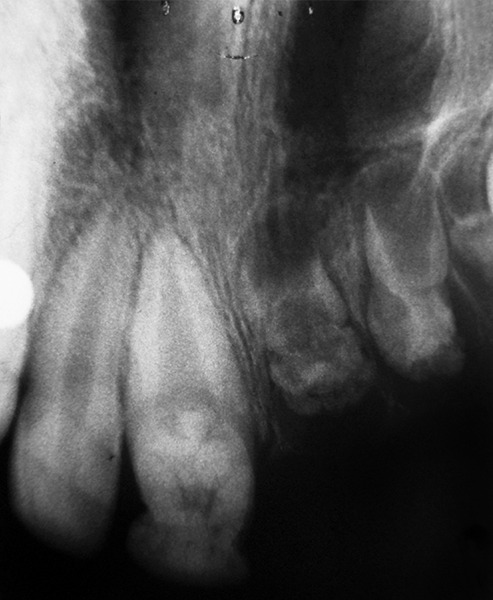
11 and 12 showed ghost-like teeth and 21 showed wide pulp chamber with malformed crown

**Fig. 4B:**
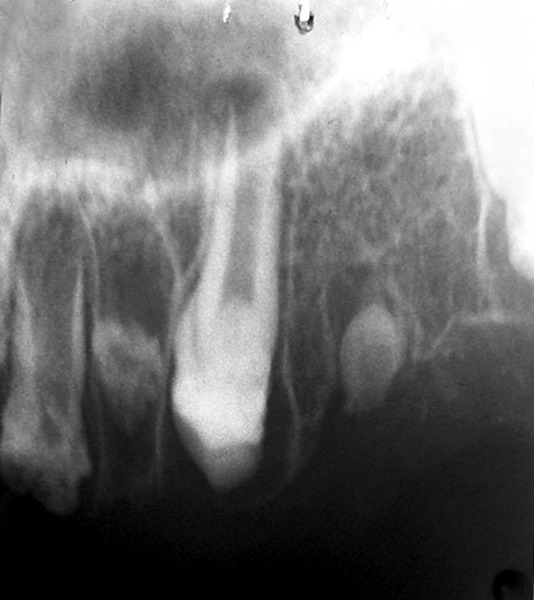
12,13,15 and 16 showed ghost-like but 14 not affcted as other

## DISCUSSION

Regional odontodysplasias are probably misdiagnosed as malformed teeth or odontomas. Other conditions, such as dentinal dysplasia, shell teeth, hypophosphotasia, dentino-genesis imperfecta or amelogenesis imperfecta can mimic some features of regional odontodysplasia.^6^ However, these disorders tend to affect the entire dentition.^4^ The criteria of diagnosis of regional odontodysplasia are primarily clinical and radiographic. Clinically affected teeth have an abnormal morphology and irregular surface contour, with pitting and groves surface. The teeth appear to be discolored, hypoplastic and hypocalcified.^7^ It is possible to find some teeth without any alterations in the affected quadrant. Affected teeth are more susceptible to caries and susceptible to fracture. Tooth eruption is delayed or does not occur. The most frequent clinical symptoms after eruption of teeth are gingival swelling, periapical infection and abscess formation in the absence of caries. Radiographically, the affected teeth show a “ghost-like” appearance due to reduced thickness and radiodensity of enamel and dentin. There is no visible demarcation between hypomineralized dentin and hypomineralized enamel.^9^ The teeth tend to be shorter, have short roots with wide open apices and abnormally wide pulp chambers and canal.^10^

Although many theories have been proposed for the development of regional odontodysplasia but its etiology was unknown. It has been proposed that an imbalance of necessary proteins might lead to the structural disorganization seen in this anomaly, such as the metalloproteinase (MMPs), which are enzymes that play a key role in dental development.^2^ Regional odontodysplasia affected only one quadrant in the maxilla, rarely crossed the midline.^5^ Regional odontodysplasia seems to be more prevalent in females as found in this case.^8^ The clinical presentation of this case was failure in eruption of teeth, which could be related to odontodysplasia. In this case, radiographs showed that the unerupted teeth had not achieved complete morphogenesis. The affected erupted (left maxillary central incisor) teeth had an abnormal morphology with an irregular surface contour with pitting and grooves, and a rough surface with defective mineralization. Radiographically, the affected unerupted teeth have been described as “ghost-like” appearance, showing a marked reduction in radiodensity. Both the enamel and dentin appear very thin, and the pulp chamber is exceedingly large.

The patient of this case report exhibited many of the common clinical and radiographic features consistent with the diagnosis of regional odontodysplasia .The clinical and radiographic characteristics involving the permanent dentition in the maxillary right quadrant (and also including the left permanent central incisor) strongly supported the diagnosis of this condition.

## References

[1]  Magalhães AC, Pessan JP, Cunha RF, Delbem AC (2007). Regional odontodysplasia: case report.. J Appl Oral Sci.

[2]  Gondim JO, Pretel H, Ramalho LT, Santos-Pinto LA, Giro EM (2009). Regional odontodysplasia in early childhood: A clinical and histological study.. J Indian Soc Pedod Prev Dent.

[3] (2006). Cho SY Conservative management of regional odontodysplasia: Case report.. J Can Dent Assoc.

[4]  Kappadi D, Ramasetty PA, Rai KK, Rahim AM (2009). Regional odontodysplasia: An unusual case report.. J Oral Maxillofac Pathol.

[5]  Tanase S, Yasui S, Kondo T, Otsuji W, Yao J, Kondo S, Suzuki Y, Nishi H, Yamada S, Ken-ei Ryuzaki (2000). Long-term treatment of regional odontodysplasia located in maxillary molars.. Japanese J Pediatr Dent.

[6]  Rosa MC, Marcelino GA, Belchior RS, Souza AP, Parizotto SC (2006). Regional odontodysplasia: report of case.. J Clin Pediatr Dent.

[7]  Ozer L, Cetiner S, Ersoy E (2004). Regional odontodysplasia: report of a rare case.. J Clin Pediatr Dent.

[8]  Crawford PJ, Aldred MJ (1989). Regional odontodysplasia: A bibliography.. J Oral Pathol Med.

[9]  Vaikuntam J, Tatum NB, McGuff HS (1996). Regional odontodysplasia: Review of the literature and a case report.. J Clin Pediatr Dent.

[10]  Cahuana A, Gonzalez Y, Palma C (2005). Clinical management of regional odontodysplasia.. Pediatr Dent.

